# Use of computational intelligence techniques to predict flooding in places adjacent to the Magdalena River

**DOI:** 10.1016/j.heliyon.2020.e04872

**Published:** 2020-09-14

**Authors:** Jenny Marcela Moreno, Juan Manuel Sánchez, Helbert Eduardo Espitia

**Affiliations:** Universidad Distrital Francisco José de Caldas, Bogotá, Colombia

**Keywords:** Earth sciences, Environmental science, Systems engineering, Control systems, Computer engineering, Climatology, Environmental economics, Artificial intelligence, Flood, Artificial Neural Networks, Neuro Fuzzy Systems, Support Vector Machine, Magdalena River, Climate variability

## Abstract

Floods are one of the worst natural disasters in the world. Colombia is a country that has been greatly affected by this disaster. For example, in the years 2010 and 2011 there was a heavy rainy season, which caused floods that affected at least two million people and there were economic losses of 6.5 million dollars, which is equivalent to 5.7% of the country's Gross Domestic Product (GDP) at that time. The Magdalena River is the most important since 128 municipalities and 43 cities with a population of 6.3 million people, which is 13% of the total population of the country, are located in its basins. For this reason, the objective of the research is to design and implement a model that helps predict flooding over the Magdalena River by examining three techniques of artificial intelligence (Artificial Neuronal Networks, Adaptive Neuro Fuzzy Inference System, Support Vector Machine), and thus determining which of these techniques are the most effective according to the case study. The research was limited only to these three types, due to limitations of time, data, human and financial resources, and technological infrastructure. In the end, it is concluded that the Artificial Neural Networks technique is a suitable option to implement the predictive system as long as it is not very complex and does not require high processing machine. However, to establish a model based on rules to achieve a better interpretability of the floods, the ANFIS model can be used.

## 1 Introduction

Floods are considered among the worst natural disasters occurring worldwide causing human and economic losses. Floods are present even in developed countries like Australia, Japan, and the USA, and in under developing countries like Indonesia, Pakistan, Colombia, and Bangladesh; however, it is the second group where the consequences are more severe, in accordance with the Annual Global Climate and Catastrophe Report that also indicates that of the years 2015 [1], 2016 [2] and 2017 [3] the most negative effects were seen in 2015 when the floods caused losses of 27 trillion-dollars around the world.

Colombia has large extensions of land which are susceptible to floods due to its topographic characteristics and hydro meteorological regimen, especially nearby parts of the rivers Atrato, Cauca, Magdalena, and Putumayo [4]. According to historical records of the data base of “DesInventar.org”, floods are among the main cause of losses in the territory [5]. Floods, landslides, gales (strong winds) and associated climatic events related to “La Niña” phenomena 2010-2011 left more than two million people affected, according to figures provided by the Registro Único de Damnificados (RUD) of the Unidad Nacional de Gestión de Riesgo (UNGR). The last event was characterized by floods that caused losses in infrastructure, agricultural goods, livestock, crops, and forests that are the source of income and consume of the local inhabitants in rural areas [6]. According to ECLAC (Economic Commission for Latin America and the Caribbean), the damages and economic losses reached 6.5 billion dollars, equivalent to 5.7 percent of the income of the country for that time.

In accordance with the annual report of the Global Climate Risk Index carried out by the Non-Governmental Organization (NGO) Germanwatch, from 2013 to 2018, Colombia is found among 50 countries most widely affected by natural disasters like hurricanes, floods, and heat waves; in addition, in the 2012 report the country ranked third place of the countries affected by the consequences of “La Niña” phenomena in 2011. [7].

Hydrological engineering aims to plan, analyze, design, build, and operate projects for the control, use and management of water resources [8]. Among the tools that can be used for this purpose, there is soft computing or computational intelligence that seek to understand natural phenomena for the development of algorithms. Among these are the genetic algorithms (GA), optimization of particle swarm (PSO), optimization of ant colonies (ACO), among others [9]. Some works related to this topic are described below. The objective of [10] was to handle the large variation issues of hydric data in fuzzy data by constructing a variable spread Multivariate Adaptive Regression Splines (MARS) via fuzzy regression model with crisp parameters estimation. Another work is that of [11], since shows how soft computing tools have helped the prediction of scouring characteristics as one of the main problems in hydrological engineering. The research [12] presented an Artificial Neuronal Networks (ANN) with modified multi-layer perceptron (MLP) model based on decision trees (DT-MLP), this method improved performance in forecasting velocity and water free-surface profiles in a 90° open-channel bend with respect to previous research. Those works can be grouped according to the implementation of Artificial Neuronal Networks (ANN), Artificial Neuronal Networks Auto-Regressive (NNAR) with exogenous inputs (NNARX), and with other additional techniques.

The works implementing ANN are characterized for its simple implementation to predict values since it is possible the generation of an effective model to solve different issues as these models have series of time organized including suitable variables. This is observed in studies carried out in [13], [14], [15], [16], and [17], where the authors demonstrate that using ANN to predict floods is adequate as the Root of the Square Mean Error (RSME) oscillates between 0.0007540 and 0.93. These studies were focused on those characteristics that might transform an artificial neuronal network into a more effective one; for instance, the analysis undertaken by Johannet et al. [18], concluded that the results were improved when having hidden layers in the network. Regarding the training algorithm, [13] concluded that the Bayesian Regularization algorithm presented a surpassing behavior. Additionally, the number of stations concur with the studies [19] and [14] in which only three measurement stations along the Kelantan and Phraya rivers were considered. There have been additional explorations aiming for the optimization of results thanks to the artificial neuronal networks. In [20] a Particle Swarm Optimization (PSO) was implemented for finding optimal parameters (number of hidden layers, number of inputs, and ranges in the levels of water) for training the ANN. The study [21] tested the suitability of the Lower Upper Bound Estimation (LUBE) method to construct ANN in producing prediction intervals at different confidence levels for the 6 hours ahead streamflow discharges of the Susquehanna and Nehalem Rivers in United States. The study concluded Multi-Objective Fully Informed Particle Swarm (MOFIPS) based LUBE represents a viable option for straightforward design of more reliable interval-based streamflow forecasting models. Meanwhile in [22] data is classified in different categories using the method k-means, focusing on the improvement of the capacity of a non-linear simulation; later, various Stacked Autoencoders with Back Propagation (SAE-BP) modules were adapted to simulate each category. This approach was compared to Support Vector Machine (SVM) models, Back Propagation artificial neuronal network, artificial neuronal network of Radial Basis Function (RBF), and Extreme Learning Machine. The results showed that the SAE-BP integrated algorithm performed exceedingly noticeable in regard of the other models. Likewise, [23] also implemented SVM models thus obtaining a better performance about the data peaks present in the sets of data. Moreover, the same study implemented an experiment using Adaptive Neuro Fuzzy Inference System (ANFIS), this allowed the reduction of noise, hence generated less disperse results among all the implemented techniques. Finally, the study [24] used several soft computing approaches were employed for rainfall prediction in the Zhenshui and Da'ninghe watersheds of China. They used preprocessing techniques included moving average (MA) and singular spectrum analysis (SSA). The modular models were composed of local support vectors regression (SVR) models or artificial neural networks (ANN) models. Results showed that the MA was superior to the SSA when they were coupled with the ANN.

A variation of artificial neuronal networks is given in NNARX. These networks grant the modeling of non-linear systems when considering exogenous inputs as in the case of the forecast of floods where variables like the volume of a flood, the amount of rain and overflows are considered. In this regard, [25] implemented data of the Kelang river to predict the level of water for three hours in advance; the same study compared four different cases in which the variation was given in terms of time; besides, different algorithms were carried out: Gradient Descent (GD), Levenberg-Marquardt (LM) and One Step Secant (OSS). The LM training algorithm was used in [26] showing a suitable performance, in [27] a comparison of its performance with that of the Bayesian Regularization was performed, while [28] found that the GD algorithm presented a better performance compared to the algorithm OSS.

Regarding the number of stations, [26] and [29] took the data of three stations in three rivers in a highly vulnerable point and where two of the three rivers converge. Regarding the exogenous inputs, overflow average and the registry of rains were applied; thus, from the total data 70% was taken to train the NNARX, and the remaining 30% to perform tests. Both, [28] and [29] comparison performances of NNARX together with the ANN and NNAR were made to confirm that the best results were obtained with the first technique.

Finally, research [30] used a deep learning data-intelligence model based on the long short-term memory, this was developed to forecast the streamflow of the Kelantan River in the northeast region of the Malaysia Peninsula. This research concluded that the developed LSTM model has both obvious advantages in processing steady streamflow data in the dry season, and a suitable ability to capture data features in the rainy season.

The above proves that it is necessary to determine new strategies for both preventions of disasters and to mitigate risks, which is the focus of this paper where a model that allows knowing the Magdalena River in advance is developed, starting with previously collected data provided by the IDEAM (Instituto de Hidrología, Meteorología y Estudios Ambientales).

The objective of the research is to design and implement a model that helps to predict the floods on the Magdalena River, examine different artificial intelligence techniques (Artificial Neural Network, Neuro Fuzzy Systems, Support Vector Machine), and thus determine the factors of these techniques that are more effective according to the case study, recognizing that each technique has certain particular characteristics. It is clarified that the development of a new algorithm is not within the scope of the research.

In a previous investigation, a preliminary study was made where the number of samples was smaller and only a few configurations of the model were tested in Artificial Neural Networks [31]. The research was limited only to these three types of techniques (ANN, ANFIS, SVM), due to limitations of time, data, human and financial resources, and technological infrastructure. The main advantage of the implementation of these soft computing techniques in this case is that they allow solving non-linear problems, in which mathematical models are not available. The reason is the high difficulty of making a mathematical model due to the complexity of the climatic variability in the tropics and especially in Colombia, as a result of the presence of a large number of microclimates and the influence of “El Niño” and “La Niña” phenomena [32].

But it is pertinent to continue with the research including other artificial intelligence prediction techniques such as machine learning, expert systems, Bayesian networks, among others, with the availability of greater resources for the development of the research. For example, a more robust computer system with which a greater number of experimental tests can be carried out.

The decision was made to hold the investigation on the Magdalena River, as it is the most important river in Colombia, due to its extension, the municipalities and population that are located in its basin, the relevance that it has within the Colombian economy and the data availability. Nevertheless, Colombia has a large number of rivers given its topography, thus, it is prudent that the results of this research be taken as a basis for future research in other rivers of the Colombian geography such as the Cauca river, which is the second most important in Colombia and other rivers of great importance such as the Bogotá, Atrato, Sinu, Patía, San Juan, Baudo, Arauca, Guaviare, Meta and many other rivers.

The data of this research was collected by IDEAM (Institute of Hydrology, Meteorology and Environmental Studies), which is a Colombian public institution of technical and scientific support; this entity allowed access at the time of the research to the data comprised from 1997 to 2015, unfortunately, the data from more recent years could not be accessed. Nevertheless, these will not have significant effects on the results because in recent years there have been no significant phenomena in the climate and the most relevant in the tropical zone are “El Niño” and “La Niña” phenomena, which are present within the data sample.

The proposed model starts from the analysis of external variables that may have an impact on the level of the river including: the location of the station of measurement, the presence of “El Niño” or “La Niña” phenomena, and the station where the event takes place. Each variable was used as input in the codification of the model. The results obtained display an acceptable behavior; however, they also show the relevance of the interpretability that is given to the output and also the way by which the phenomenon is generated and its causes.

The article is organized as follows: the first sections include the theoretical framework of the techniques considered; then, there is a description of the methodology. Later, the results obtained are displayed, finally, the section of conclusions that include a discussion.

## 2 Magdalena River

Hydrometric provides the data related to the temporary and spatial distributions of the water on the ground, this allows determining the hydrological conditions that can cause a flood. The ground already contains the tools that provide the information on the volume of water, that is, the amount of fallen rain; these are called rain gauges. As to those that provide information about the water flow increase, there are the called hydrometric scales [33].

**Figure 1:**
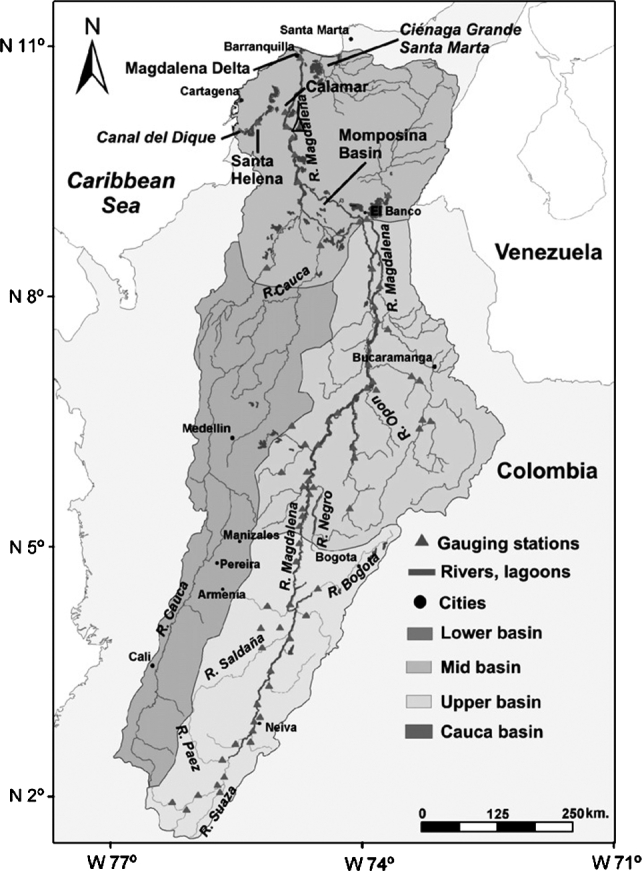
Magdalena River. Source [36].

The Magdalena River is born in the Lake La Magdalena in the Paramo de Las Papas, Huila at 3.685 meters above the sea level, pouring its waters in the Caribbean sea in Bocas de Ceniza, whose volume of water at this point is 6.700 m^3^/s [34]. Its length is 1.540 Km approximately and is considered as the main river artery as this river crosses 22 departments of the country [35], including Magdalena, Atlántico, Barranquilla, Bolívar, Cesar, Antioquia, Santander, Boyacá, Cundinamarca, Caldas, Tolima, and Huila, as presented in Fig. 1.

In its watershed, there are 128 riverside municipalities and 43 cities that in turn are ports [37]. There live 6.3 million people, which is 13% of the Colombian population by the end of 2017 [38]. The hydrological regimen of Magdalena River presents a seasonal character since there is an increase of flows due to the high rainfall in its watershed; this produces the riverbed be exceeded and producing overflows toward the swamps and the Canal del Dique. Such regimen has changed through time for physical elements and for human action far and wide the watershed, deforestation, mismanagement of soils and the river basin that have caused variations in the volume of flow that make the affected areas as of high risk [39]. The lowest water levels are registered in January, February, March, July, August, and September. Whatever the conditions, it is relevant to indicate that the lack of adequate and preventive management of the river flow becomes natural disasters either floods or droughts with the socioeconomic consequences above mentioned.

## 3 Artificial intelligence

This term was first coined in 1956 by the scientists McCarthy, Minsky, Rocherster, and Shannon who defined it as “the science and the engineering to create intelligent machines” [40]. On the other hand, for Poole and Mackworth AI is about systems acting rationally, more specifically, as the “study of the design of intelligent agents” [41]. An agent is an entity acting in a specific environment that performs actions according to the goals proposed. Other characteristics are the flexibility in a changing environment, learning acquired from experience, and its capacity in decision-making processes based on perceptive and computational limitations [41].

### 3.1 Machine Learning

Machine Learning is a mechanism used to search patterns and develop intelligence in a machine, in order to be able to learn, which implies that it will have the ability to perform better in the future from its own experience [42]. Data is a key factor in the Machine Learning field, some important concepts are:

• Attribute or variable: column of data that is referenced in the learning algorithm. These attributes can be inputs or outputs of the algorithm.
• Instance: a single row of data in the dataset.
• Variable vector: list of variables.
• Dimension: subset of attributes used to describe a property of the data. For example, the “Date” dimension consists of three attributes: day, month, and year.
• Dataset: collection of rows or instances of data. There are different types of datasets, which are used for different purposes: training, testing and evaluation. Normally, a dataset is divided into the following proportions: 60% training, 30% tests and 10% evaluation.
• Training dataset: data with which the model is built or trained.
• Test dataset: data that allows validating the built model.
• Evaluation dataset: data used for the final verification of the model.

### 3.2 Artificial Neural Networks

Artificial Neural Networks (ANN) are defined as information processing systems whose structure and operation are inspired by biological neural networks; they consist of a set of simple processing elements called nodes or neurons, which have connections with an associated weight [43].

**Figure 2:**
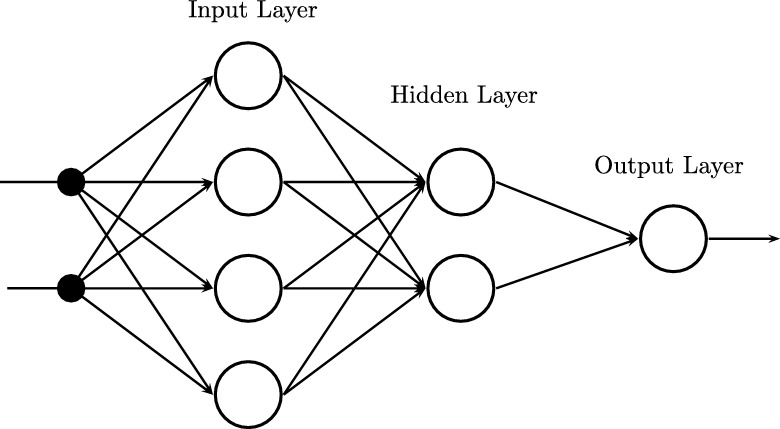
Multilayer Neural Network.

The multilayer perceptron is one of the most representative Artificial Neural Networks, showed in Fig. 2. Regarding the development of ANN in [44] is shown that the combination of several perceptrons (inclusion of hidden neurons) can be a solution for different nonlinear problems. Thus, [45] presents a form of backpropagation of the errors measured at the output of the network towards the hidden neurons, corresponding to the general delta rule [45]. Additionally, the multilayer perceptron provides a structure to relating input and output variables, a relationship that is provided by propagating the values of input variables towards the next layer of the network until the respective values are obtained in the outputs [46].

#### 3.2.1 Recurrent Networks

Recurrent Artificial Neural Networks (RNN) are a type of ANN that have feedback connections between the elements. A neuron is connected to posterior neurons and also with the neurons of the previous layer. In this way, this type of neural network can handle sequences of data that vary in the time domain [47].

**Figure 3:**
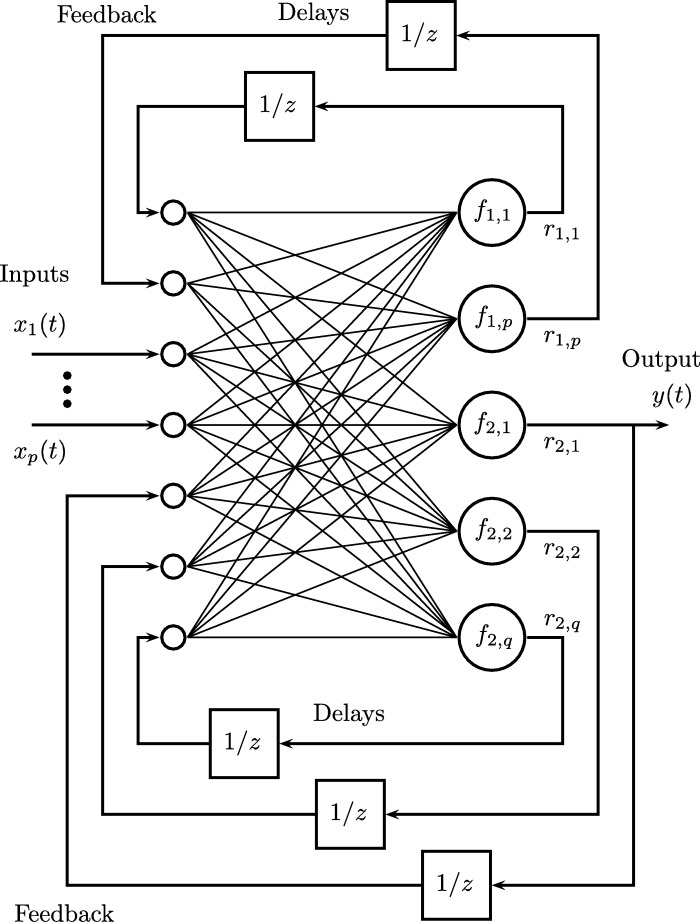
Recurrent neural network.

The RNNs (Fig. 3) are especially useful for predicting events based on previous inputs. In its architecture the inclusion of delays at the output of neurons in intermediate layers can be observed. The partial outputs $r_{mn}(t+1)$ are converted to values $r_{mn}(t)$, in this way the information stored of previous moments of the network are feedback. According to [48], Recurrent Artificial Neural Networks are an effective technique for solving problems with nonlinear time series.

#### 3.2.2 Autoregressive Artificial Neural Networks with Exogenous Inputs

Autoregressive Artificial Neural Networks with Exogenous Inputs (NNARX) are dynamic recurring networks that are based on the current value of a series y(t) that can be a function of the previous values of the same variable [49].

**Figure 4:**
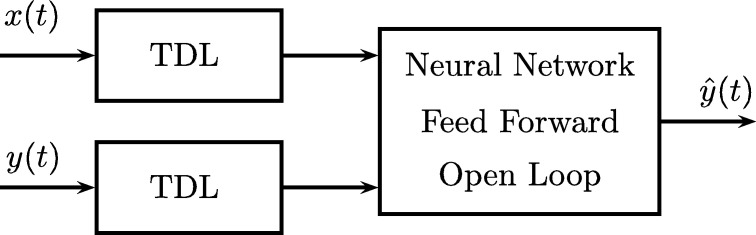
Open loop feedforward neural network.

**Figure 5:**
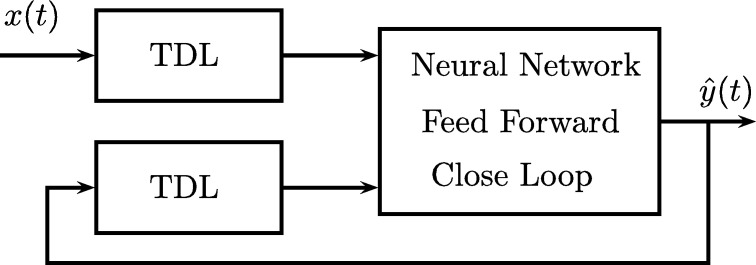
Neural feedforward network with closed loop.

As shown in Figs. 4 and 5, the NARX model can have two types of architecture, the first is the series-parallel series or “open-loop” and the second is the parallel architecture or “closed-loop”.

In the first, the future value y(t+1) of a time series is predicted from the past and present values of x(t) and the past values y(t) of the series, the following equation describes it:

(1) $$\overset{ˆ}{y}(t+1)=F(y(t),y(t-1),...,y(t-n_{y}),x(t+1),x(t),x(t-1),...,x(t-n_{x}))$$

While in the second architecture, the prediction is made using the past and present values of x(t) and the past values of $\overset{ˆ}{y}(t)$ that were predicted by the network, the following equation describes it:

(2) $$\overset{ˆ}{y}(t+1)=F(\overset{ˆ}{y}(t),\overset{ˆ}{y}(t-1),...,\overset{ˆ}{y}(t-n_{y}),x(t+1),x(t),x(t-1),...,x(t-n_{x}))$$

### 3.3 Neuro Fuzzy Systems

**Figure 6:**
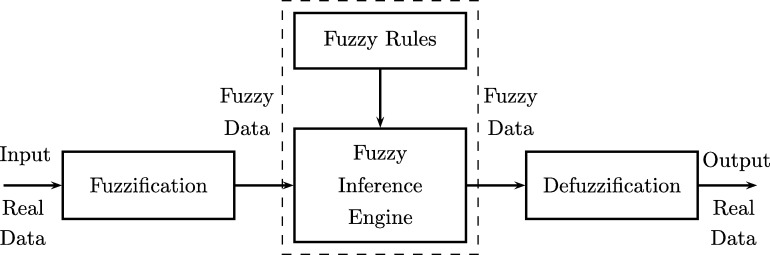
Representation of an Fuzzy Inference System.

Fuzzy systems were developed as an adaptation of fuzzy set theory, which allows the treatment of inaccurate or uncertain information for the modeling and control of complex systems [50]. By using rules with fuzzy antecedents and consequent to handle uncertainty it is possible to build models close to human reasoning. This type of systems allows applications in automatic control, data classification, decision analysis, expert systems, time series prediction, robotics and pattern recognition [51]. A Fuzzy Inference System FIS (Fig. 6) has the following elements [52]:

1. A knowledge base formed by a base of fuzzy rules and membership functions of fuzzy sets used in fuzzy rules.
2. A reasoning mechanism that performs operations over the fuzzy rules.
3. A fuzzifier or fuzzy adaptation process, which allows transform the input into fuzzy values.
4. A defuzzifier or simple adaptation process. It allows to transform a result fuzzy set into a representative real value.

#### 3.3.1 Adaptive Neuro Fuzzy Inference Systems

Adaptive Neuro Fuzzy Inference Systems (ANFIS) were proposed in 1993 by Roger Jang [53], who combined fuzzy systems and Artificial Neural Networks, taking advantage of the intrinsic advantages of each of these techniques. The ANFIS allow to represent knowledge in an interpretable way and also have the ability to train and learn, which greatly improves the performance of the system [54]. Usually, for implementing ANFIS is used the fuzzy model Takagi-Sugeno-Kang (TSK).

**Figure 7:**
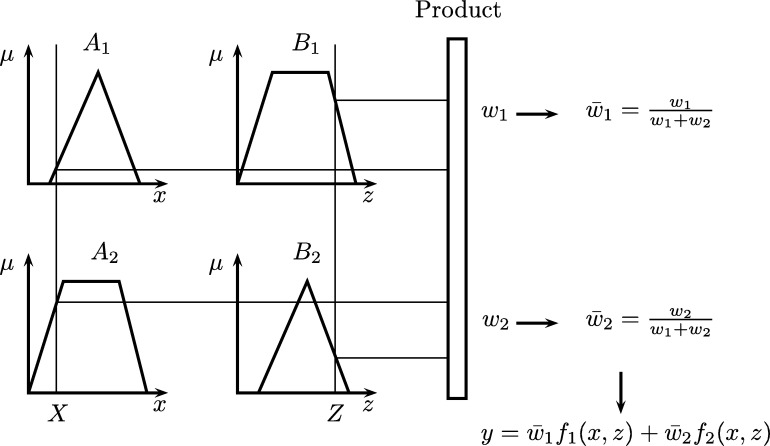
Graphical representation of the model Takagi-Sugeno.

**Figure 8:**
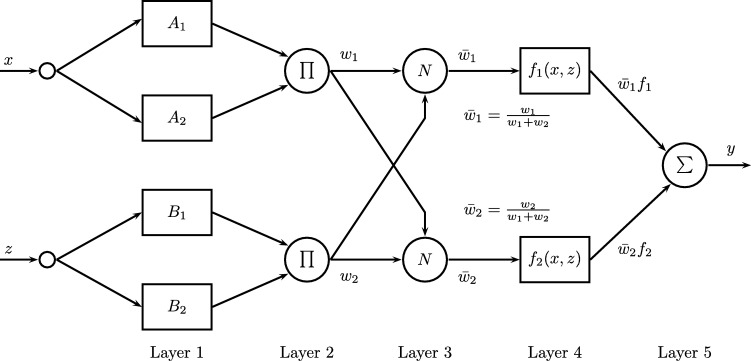
Scheme of an ANFIS TSK.

The representation of the Takagi-Sugeno model can be seen in Fig. 7. This system can be interpreted as a feedforward neural network, as shown in Fig. 8.

Each layer has several nodes, being those that are represented with rectangles (layers 1 and 4) which can be adapted and those represented by circles (layers 2, 3, and 5) are fixed.

Assuming that ANFIS has two rules, their respective equations can be described as:

• **Rule 1:** If *x* is $A_{1}$ and *z* is $B_{1}$, then $f_{1}=p_{1}x+q_{1}z+c_{1}$.
• **Rule 2:** If *x* is $A_{2}$ and *z* is $B_{2}$, then $f_{2}=p_{2}x+q_{2}z+c_{2}$.

In the first layer, each node establishes the degrees of membership of a linguistic variable and implements a fuzzification. The function is described in the equation (3). Where *x* corresponds to the node input *i*, and $A_{i}$ is the linguistic variable associated with the node.

(3) $$o_{i}^{1}=\mu A_{i}(x)$$

In the second layer, the activation weight of the rules is calculated in the nodes and the result is transmitted, as stated in equation (4).

(4) $$w_{i}={\mu_{A}}_{i}(y)\times {\mu_{B}}_{i}(y),i=1,\;2$$

Each output node represents the activation weight of a rule. The third layer normalizes membership values by calculating the proportion of the activation weight of the *i*-th rule with respect to the sum of the activation weights of all rules (equation (5)).

(5) $$w_{i}=\frac{w_{i}}{\left(w_{1}+w_{2}\right)},i=1,\;2$$

In the fourth layer, the product is calculated between the result of the normalized values and the functions that depend on the input. For this the equation is used (6).

(6) $$o_{i}^{4}=w_{i}(p_{i}x+q_{i}z+c_{i})$$

where $w_{i}$ is the output of layer 3 and $p_{i}$, $q_{i}$ and $r_{i}$ are the set of parameters.

Finally, the last layer calculates the general output, using the equation (7).

(7) $$o_{i}^{5}=\sum_{i}{\bar{w}}_{i}f_{i}=\frac{\sum_{i}w_{i}f_{i}}{\sum_{i}w_{i}}$$

### 3.4 Support Vector Machine

Support Vector Machine (SVM) are a set of supervised learning methods that were originally designed to solve linear classification problems, however, they are also used in regression problems (Support Regression Vector) [55]. They were introduced in the 90s by Vladimir Vapnik in his studies on the theory of statistical learning [56]. This tool allows to represent the knowledge learned through the most informative points, called “support vectors”, used to perform the classification and also data regression. On the other hand, one of its advantages compared to ANN is the possibility that SVMs give to solve a problem from a small number of data [57].

Considering $y_{t}$ a series of time, with regressors $x_{t}$, and *D* representative examples; an SVM allows to approximate the value of the variable $y_{t}$ through the following function:

(8) $$y_{t}=b+\sum_{d=1}^{D}W_{d}\times k(x_{t},x_{d})$$

Where *b* is a constant and $W_{d}$ are the weighting factors of the kernel function k(.,.). Thus, an SVM is a linear combination of the mapping of $x_{t}$ in a space defined by the points $x_{d}$ and the nonlinear transformation function k(.,.) [55].

#### 3.4.1 Kernel functions

In equation (8) the kernel function is described by $k(x_{t},x_{d})$, this function allows taking the point *x* and represents it in a space parameterized by the points $x_{d}$. There are several functions that can be used as kernels:

• Linear.
• Polynomial.
• Gaussian or Radial Basis Function (RBF).
• Exponential.

#### 3.4.2 Variable estimation

The estimation of the value of the equation (8) it is based on the minimization of the function R(C,ϵ), defined as:

(9) $$R(C,\epsilon )=C\frac{1}{D}\sum_{d=1}^{D}L\epsilon (y_{d},{\overset{ˆ}{y}}_{d})+\frac{1}{2}\sum_{d=1}^{D}W_{d}^{2}$$

Where the first part corresponds to the error between the data predicted by the model and the real data, while the second corresponds to the regularization component. For interpolation problems, the value of this function corresponds to the pre-established accuracy of interpolation, then if the value of this function is higher the number of support vectors needed are low. Normally cross-validation techniques are used to find a value for the constant *C* that does not generate an over-fitted model [58]. In order to allow some noise level, the error condition can be relaxed between the value predicted by the function and the real value, using the loss function *ϵ*-insensitive, $L_{\epsilon }$ which is a linear function with an insensitive zone width 2*ϵ*, where the error is null and is defined by the following equation:

(10) $$L_{\epsilon }(y_{d},{\overset{ˆ}{y}}_{d})=\left\{ \begin{array}{cc} |y_{d}-{\overset{ˆ}{y}}_{d}|-\epsilon ,\; & \text{if}\;|y_{d}-{\overset{ˆ}{y}}_{d}|>\epsilon \\ 0,\; & \text{otherwise.} \end{array}\right.$$

**Figure 9:**
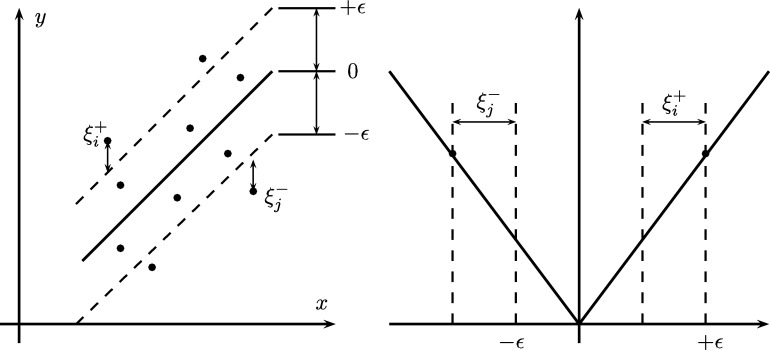
Relationship between slack variables and the *ϵ*-sensitive zone.

This feature allows a dispersion in the linear regressor solution. The values fall within the region defined by ±*ϵ* are discarded to be support vectors, thus the number of possibilities is considerably reduced. In addition, the slack variables: $\xi_{i}^{+}$ y $\xi_{j}^{-}$ are defined to know the value of the error as can be seen in the Fig. 9 [58].

## 4 Methodology

**Figure 10:**
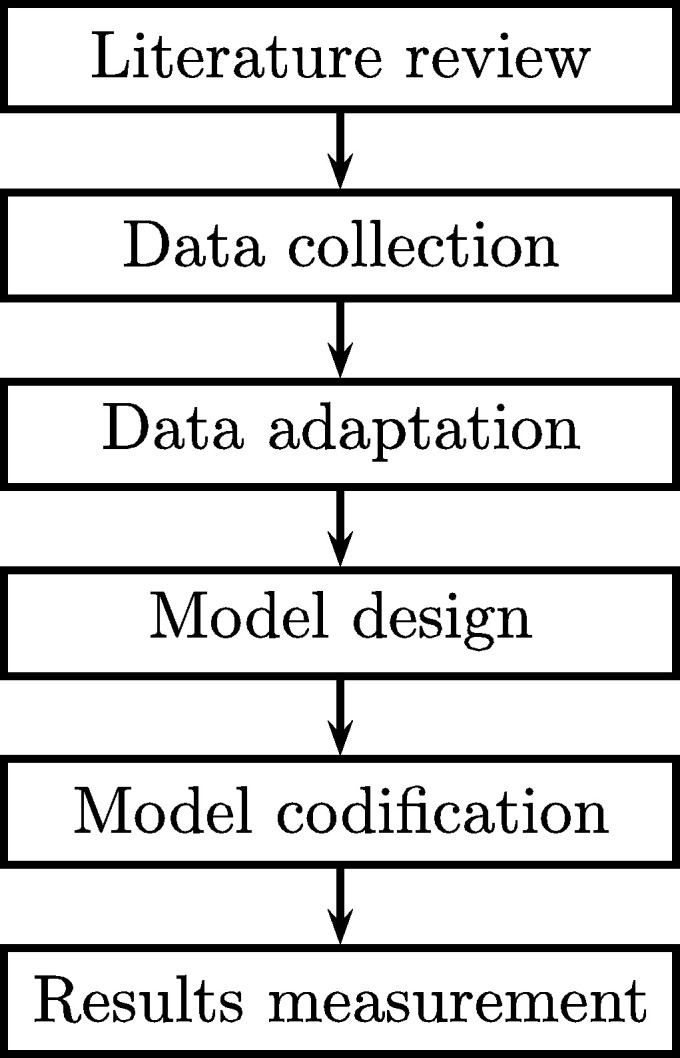
Diagram of the methodology used.

Fig. 10 presents the methodology used to develop the prediction system, which contains a flow of developed stages.

### 4.1 Conceptual framework

To carry out this work, a review of papers related to flood prediction through ANFIS, ANNs and/or NNARX, was first done to know the background in the use of these techniques. From the literature revised it is appreciated that these techniques are very useful to generate this type of models. In this regard, the question is if these techniques are useful to predict the floods of the Magdalena River.

### 4.2 Data collection

The data was provided by the institute of hydrology, meteorology and environmental studies IDEAM (Instituto de Hidrología, Meteorología y Estudios Ambientales). The variables collected were maximum, medium, and minimum water level, daily. The data range is between January 1997 and December 2015; for a total of 5 stations of the Magdalena River which are:

1. Aguadas.
2. Barbosa.
3. El Banco.
4. Palenquito.
5. Puerto Berrío.

### 4.3 Data adaptation

Because the data format delivered by the IDEAM could not be used directly, it was necessary to modify its structure using Excel macros. Once a standardized format was taken, the empty spaces and the data out of range were reviewed. To resolve these inconveniences, for each empty space a value is calculated by interpolating the closest values.

### 4.4 Model design

In order to establish the prediction model, variables such as location, presence of the phenomenon of “El Niño”, season and time series delays were taken into account. According to literature review it was determined the use of 70% of the data for model training and the other 30% for testing. Using these data percentages can be seen that the overfitting is not strong since the value of the objective function using the validation data shows a similar value and in some cases better than that obtained with the training data [59]. For a complementary study, other techniques to segment the data could be considered.

### 4.5 Prediction model inputs

For the elaboration of the model, variables related to the level of the river in a given station as well as the season of the year and the presence or absence of “El Niño” or “La Niña” phenomena were taken into account. In this way, we seek to relate these variables to the development of a possible flood. The model considers the following inputs:

• Signal: time series of river level.
• Delays: time series with signal delay for each station.
• Station: place where the data was taken. It can take different values depending on the location of the station with respect to the origin of the river. Stations will be assigned numbers from 1 (the nearest) up to 5 (the furthest).
• Season (quarter of the year): Division of the year in quarters. A value is assigned to each of them:
  1: Strong summer: December, January and February.
  2: Weak summer: June, July and August.
  3: Weak winter: September, October and November.
  4: Strong winter: March, April and May.
• Meteorological phenomena: Indicates presence of Niño or Niña phenomenon. Its possible values are:
  1: presence of Niño phenomenon.
  0: No weather phenomena.
  -1: presence of Niña phenomenon.

**Figure 11:**
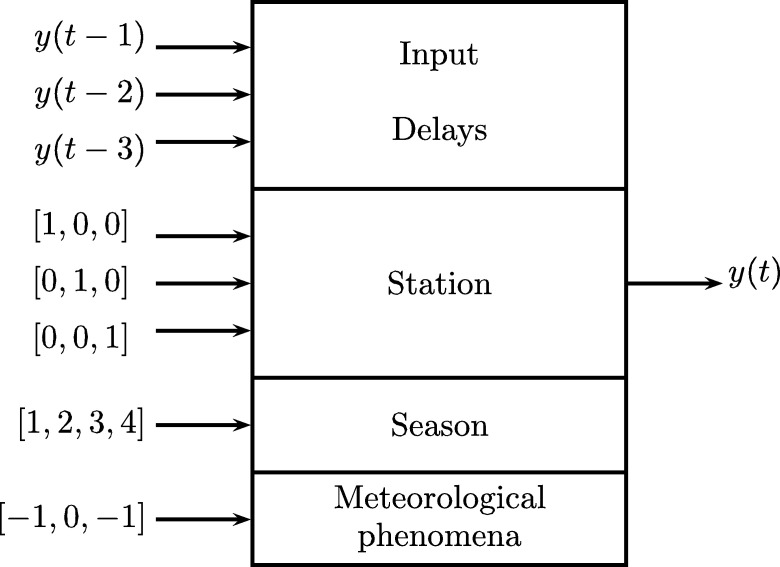
Prediction system model.

The “Station” data is represented by a number to identify each station with respect to the origin of the river. Regarding the variables of meteorological phenomena, this indicates the presence of “El Niño” or “La Niña” phenomena or the absence of both (see Fig. 11).

As observable, it is possible have different configurations of the system prediction varying the number of delays from 2 to 4. It is also important to point out that in the case of a single station, the input “Station” is not used, in this way it can have the settings shown in the Table 1.

**Table 1:** General model configurations.

Amount of stations	Delays	Total inputs
1	2	4
1	3	5
1	4	6
2 to 5	2	5
2 to 5	3	6
2 to 5	4	7

### 4.6 Results performance index

In order to measure the effectiveness of the prediction model, it is necessary to compare the predicted values versus the observed values, for which, initially the absolute error could be calculated, which is the predicted value $f_{i}$ minus the observed value $y_{i}$, $\left(f_{i}-y_{i}\right)$. With the absolute error in a time series as in this case, the error can be calculated through the performance index of the mean absolute error, which only consists of the average of all the absolute errors, for which, the total of the absolute errors, it is then divided by the number of samples. The disadvantage of this index is that the values obtained in each sample can be positive and negative, then when doing the summation it is likely that the result close to 0 will occur despite that the magnitudes of the errors are high. For this reason, this index is not used in this case, the relative error consisting of the predicted value $f_{i}$ minus the observed value $y_{i}$ could be used, all on the observed value $y_{i}$, $(f_{i}-y_{i})/y_{i}$ but this one also has the same drawback of positive and negative values. A relevant aspect to see through has to do with the difference in magnitude between the predicted and the observed value, regardless of its sign, the solution is to calculate the absolute error and square it, with which all the calculated errors are positive and thus when obtaining the average eliminates the possibility of obtaining the value 0, although the magnitudes of the errors are high. By adding the absolute errors to the table, the mean square error (MSE) indices, the root mean square error (RMSE) and the $R^{2}$ can be obtained. The RMSE index is the square root of the MSE, and the $R^{2}$ which is a normalized version of the MSE. To test the forecasting capacity of the model, the MSE index was used as it is the most widely used in the literature of the area and in this way it is easily comparable with other investigations, the $R^{2}$ was used at the time of training to fit the model, which is very useful for this purpose. The MSE and $R^{2}$ indices are described below.

#### 4.6.1 Mean Square Error

A useful measure of goodness or closeness of an estimator is called the mean-squared error of the estimator [60]. The measure of the Mean Square Error (MSE) is represented in equation (11). The MSE calculates the average of the squared value of the difference between the estimated values $f_{i}$ and the observed values $y_{i}$ where *N* is number of total data [61].

(11) $$MSE=\frac{1}{N}\sum_{i=0}^{N}{\left(f_{i}-y_{i}\right)}^{2}$$

To support the contention that the MSE of an estimator is a measure of goodness, one merely notes that (11) is a measure of the spread of $f_{i}$ values about $y_{i}$, just as the variance of a random variable is a measure of its spread about its mean. According to [60], a suitable performance index is the MSE. When of having different models a better performance is associated to small MSE.

#### 4.6.2 Performance metric $R^{2}$

The performance index $R^{2}$ can be calculated as shown by the equation (12), where the MSE(model) is the MSE seen in the equation (11), and the MSE(baseline) uses the structure of the MSE equation (11), with the difference that not each of the observed values $y_{i}$ is used, but rather the average of the observed values $\bar{y}$.

(12) $$R^{2}=1-\frac{MSE(model)}{MSE(baseline)}$$

In equation (13), the MSE(baseline) corresponds to the reference MSE as the obtained with the simplest model. The simplest possible model would be to always predict the average of all samples. A value close to 1 indicates a model with an error close to zero, and a value close to zero indicates a model very close to the simplest model. When $R^{2}$ is negative, it means that the model is worse than predicting the mean. For this reason, the $R^{2}$ index is one of the most used for the model optimization process.

(13) $$R^{2}=1-\frac{\frac{1}{N}\sum_{i=0}^{N}{\left(f_{i}-y_{i}\right)}^{2}}{\frac{1}{N}\sum_{i=0}^{N}{\left(f_{i}-\bar{y}\right)}^{2}}$$

## 5 Results

This section shows the results obtained for each implemented technique, where the MSE obtaining for training and validation of the respective configuration is stored. This value allows observing which configuration was the most effective for water level prediction. The lower value of the MSE corresponds to a more accurate model. In order to perform the experiments, the machine used has 6 GB of RAM, 500 GB of hard disk and a Core i5 processor with a frequency of 1.80 GHz. The software used to implement the models was MATLAB®, since its frequent use for this type of applications in the literature reviewed, in addition to the simple generation of graphics that allows a better results analysis. For future developments in research in the area, the Python programming language could be used. This is a multi-paradigm programming language, since it supports different types of paradigms such as object-oriented, imperative programming, and functional programming. This would allow the integration of different simulation environments achieving a greater robustness of the model [62].

### 5.1 Results using Artificial Neural Networks

**Figure 12:**
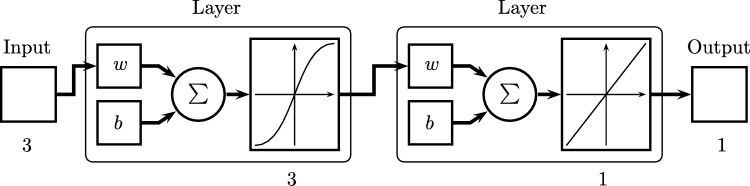
Example of case 1 of neural network.

As reference, a preliminary test of Artificial Neural Networks (ANN) model was carried out in [31]. For training and validating the model, 70% and 30% of the data was used and normalized for each purpose, and they were normalized. Data vectors were defined for each input and output for both training and testing, upper and lower limits were established for the inputs. All inputs of the model are considered a matrix. For the configurations to be tested, 3, 6, and 12 neurons were defined, and executed 30 times for each of them (because of the random initialization of Artificial Neural Networks parameters). Fig. 12 shows the first Artificial Neural Network experimented, this has three inputs and one output, in terms of data processing, it uses two layers, the first with a sigmoidal function, the second with a linear function, and 6939 data samples, this selection is based on [63] and [64]. It should be noted that for the cases of a single station, the variable associated with the type of station is not used.

According to [63] the training algorithm used was Levenberg-Marquardt and 250 training epoch were employed; likewise, 30 executions tests were performed for each configuration; once the processing was finished, the maximum (Max), minimum (Min), average (Mean) and standard deviation (STD) error were calculated, which allows to know the data dispersion. Table 2 lists the cases according to the number of stations, inputs, and neurons. In this way, different configurations are considered including the most important variables in the prediction model in order to establish the structure of the Artificial Neural Network [65].

**Table 2:** Configuration cases using neural networks.

System configuration		ANN configuration		
Stations	Inputs	3 Neurons	6 Neurons	12 Neurons
1	4	Case 1	Case 2	Case 3
1	5	Case 4	Case 5	Case 6
1	6	Case 7	Case 8	Case 9
2	5	Case 10	Case 11	Case 12
2	6	Case 13	Case 14	Case 15
2	7	Case 16	Case 17	Case 18
3	5	Case 19	Case 20	Case 21
3	6	Case 22	Case 23	Case 24
3	7	Case 25	Case 26	Case 27
4	5	Case 28	Case 29	Case 30
4	6	Case 31	Case 32	Case 33
4	7	Case 34	Case 35	Case 36
5	5	Case 37	Case 38	Case 39
5	6	Case 40	Case 41	Case 42
5	7	Case 43	Case 44	Case 45

Table 3 presents the best five cases, one for each number of stations used, finding that the best configuration is case 27, which corresponds to 3 stations, 7 inputs, 12 neurons. It is important to highlight that in 4 of the 5 cases, the best case occurred when using 12 neurons.

**Table 3:** Better results with Artificial Neural Networks.

Configuration				Min MSE	
Case	Stations	Inputs	Neurons	Training	Validation
6	1	5	12	6,79E-05	6,51E-05
12	2	5	12	4,65E-05	4,97E-05
27	3	7	12	3,60E-05	**4,77E-05**
33	4	6	12	4,12E-05	9,19E-05
41	5	6	6	4,41E-05	7,70E-05

**Figure 13:**
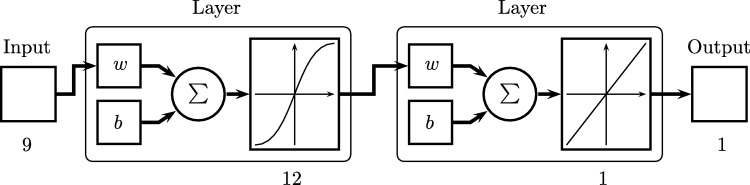
Neural network structure for 3 stations, 7 inputs and 12 neurons.

Fig. 13 presents the structure of the network that corresponds to the best case of configuration with 7 inputs, 1 hidden layer with 12 neurons and 1 output.

**Figure 14:**
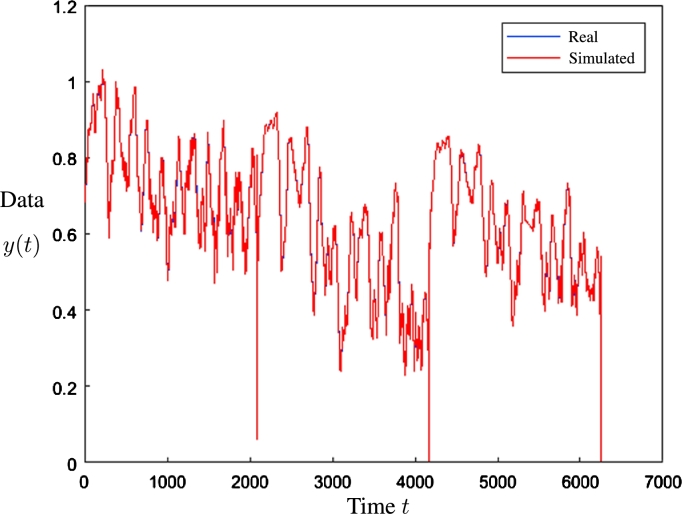
Prediction result using the Artificial Neural Network of Fig. 13.

The red line corresponds to the simulated data, which is in the foreground of Fig. 14. The background shows the blue line that corresponds to the real data. Therefore, the blue points in Fig. 14 correspond to the data when the simulation did not coincide with the real data. The same is presented in Fig. 17 for ANFIS, and 18 for SVM.

Regarding the result of the best case (Fig. 14) it is observed that in general a suitable precision was obtained, except for some values out of range (with an approximate sample of 2000, 4000 and 6000), which are extreme values.

### 5.2 Results using ANFIS

Considering [66], to perform the implementation with ANFIS a TSK fuzzy inference system is built using a “grid partition” to generate the membership functions by dividing the ranges of the input variables evenly. The fuzzy rule base contains a rule for each combination of input membership functions. Afterwards, according to [66] the training is carried out using a combination of the least squares gradient descent and backward propagation methods. Finally, the evaluation of FIS was performed obtaining the respective output for each input combination.

**Figure 15:**
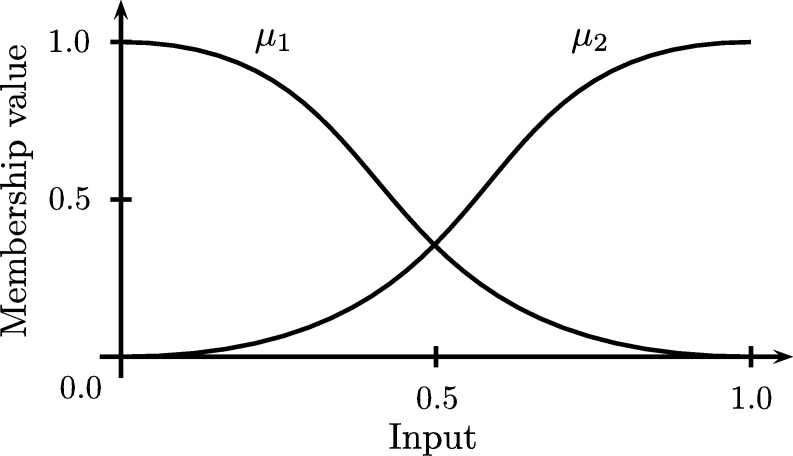
Fuzzy sets associated to input 3 of the model.

**Figure 16:**
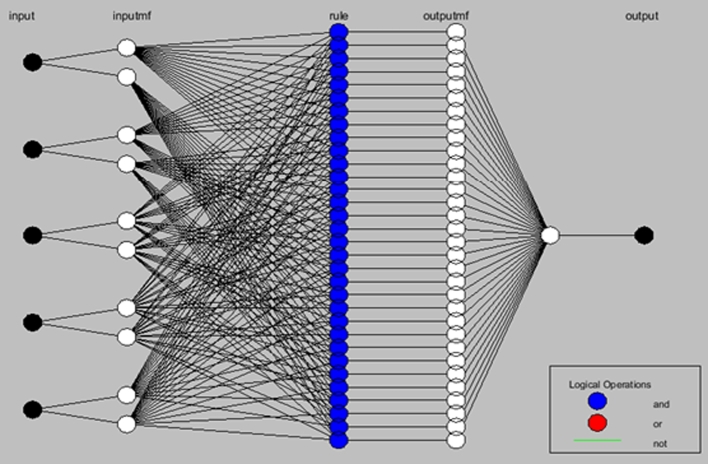
ANFIS structure for the case of one station and five inputs.

The membership function configuration of input number three can be seen in Fig. 15, this membership functions allow to describe low and high input values. The configuration cases and the respective training and validation results are presented in the Table 4. In this case, since there is a previous initialization of the fuzzy sets, it is only necessary to carry out a training process completing all the training epochs. Some cases where the rules increase considerable could not be processed after 6 hours, thus in the results table are marked using a hyphen (-). In this way the lower MSE corresponds to the better configuration in terms of prediction accuracy. As example, considering the case of one station and five inputs, Fig. 16 shows the ANFIS structure with a total of 32 rules.

**Table 4:** MSE results for training and validation.

Configuration		MSE	
Stations	Inputs	Training	Validation
1	5	6,72E-05	2,80E-03
1	6	6,68E-05	5,30E-03
2	5	4,39E-05	2,90E-03
2	6	4,35E-05	1,30E-03
2	7	-	-
3	5	3,67E-05	**1,77E-04**
3	6	3,38E-05	1,60E-03
3	7	-	-
4	5	3,84E-05	1,30E-02
4	6	3,81E-05	3,00E-03
4	7	-	-
5	5	4,03E-05	1,40E-03
5	6	3,99E-05	3,80E-03
5	7	-	-

**Figure 17:**
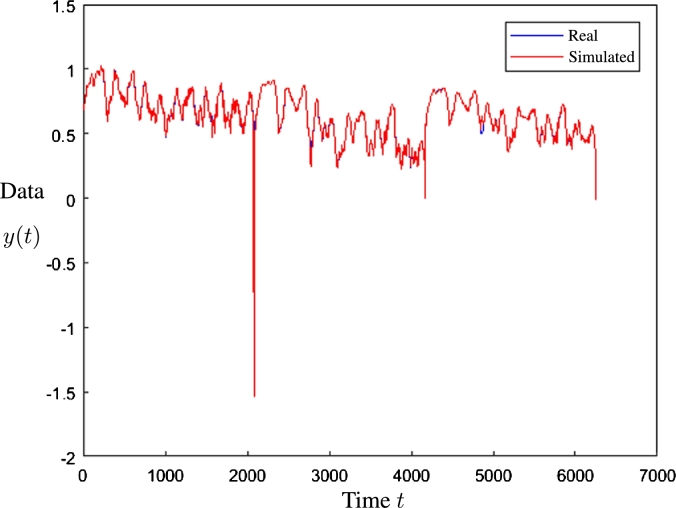
Prediction results using ANFIS.

The data of Table 4 indicate that the configuration with the best performance was that of 3 stations and 5 inputs with a MSE of 1.77E−04; on the other hand, the worst performance was that of 4 stations and 5 inputs with an error of 1.30E−02. In Fig. 17, the real and simulated data in the validation stage are presented for the best case obtained. It is possible to observe that the values overlap in most points, which indicates the precision in the forecasting process.

### 5.3 Results using Support Vector Machine

To perform the implementation of SVM first the respective normalization was performed and later, the cross-validation that allowed to select the type of kernel that would give better results. For this purpose, the parameter of cross-validation partition was used with k=5, which means that the data was randomly divided into 5 parts, the model was trained with 4 of them and validated with the rest. The above was executed for the three options available: Linear, Gaussian and Polynomial [67], [68].

The training process is performed to obtain the MSE of each model, yielding the results presented in Table 5; according to this record, the kernel that generates a better prediction is linear.

**Table 5:** Value of MSE according to the kernel.

Kernel	MSE
Linear	4,8831E-05
Gaussian	7,9302E-05
Polynomial	1,6245E-04

Once the regression model was trained the predicted values of the model were calculated. Besides, the error measurement is stored corresponding to the MSE between the values predicted by the SVM model and the actual values [67].

In total, 14 different configurations for this technique were validated. The lowest MSE corresponded to 4 stations and 5 inputs with an error of 5.07E−05. The results are presented in Table 6.

**Table 6:** Values of MSE using SVM.

Configuration		MSE	
Stations	Inputs	Training	Validation
1	5	4,37E-05	8,30E-05
1	6	3,12E-05	8,27E-05
2	5	2,94E-05	6,19E-05
2	6	3,77E-05	6,10E-05
2	7	3,96E-05	6,14E-05
3	5	2,65E-05	5,25E-05
3	6	2,36E-05	5,23E-05
3	7	3,20E-05	5,50E-05
4	5	3,71E-05	**5,07E-05**
4	6	2,91E-05	5,14E-05
4	7	2,59E-05	5,38E-05
5	5	2,91E-05	5,24E-05
5	6	2,33E-05	5,19E-05
5	7	1,73E-04	2,11E-04

**Figure 18:**
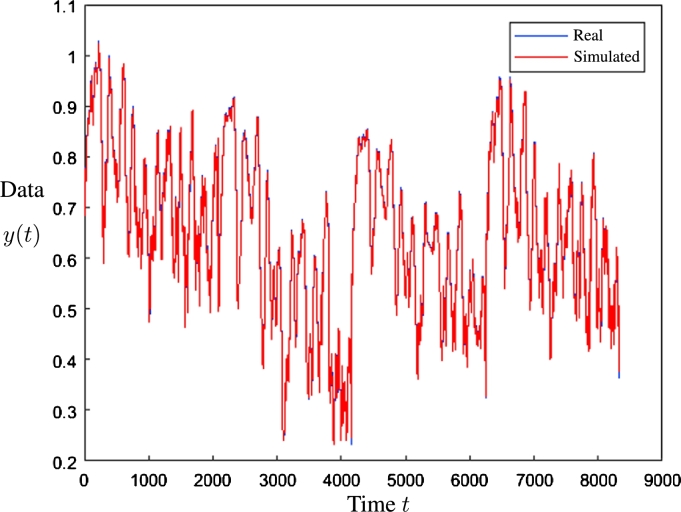
Simulation results using SVM.

In general, it can be seen that the lowest average error was recorded for the configurations for 4 stations, while the highest corresponds to those related to 5 stations. Considering the best case, a suitable level prediction is shown in Fig. 18.

## 6 Discussion

Given the stochastic nature for ANN and non-stochastic feature for ANFIS and SVM, it is difficult to make a statistical comparison via ANOVA or even Kruskal Wallis test, however, a direct comparison is presented for the best cases obtained. This corresponds to the general results of the implemented techniques.

Table 7 shows the best results obtained with the 3 techniques applied (Artificial Neural Networks, ANFIS, and Support Vector Machine).

**Table 7:** Best MSE values per technique.

Technique	Configuration	MSE
Artificial Neural Networks	3 stations, 7 inputs, 12 neurons	4,77E-05
ANFIS	3 stations, 5 inputs	1,77E-04
Support Vector Machine	4 stations, 5 inputs	5,07E-05

Under the above consideration, it is observed in Table 7 that the technique with the best result was Artificial Neural Networks, however, to obtain a model based on rules that contain the information on the relationship between the variables that allow predicting flooding, the ANFIS model would be the best option. To achieve a better interpretability, it would be convenient to convert the TSK fuzzy model to a Mamdani where there are fuzzy sets at the inputs and outputs. An additional aspect to consider is to note that the ANFIS models presented the worst performance as well as the training of all the models was not achieved.

In order to carry out a compact and representative experimental design, the configurations of the techniques used were based on the references [31], [63], [64], [65], [66], [67] and [68] In this way, the experimental was limited given the computational restrictions, which was observed when training neuro-fuzzy systems. For other works, a more extensive experimental design could be carried out considering more inputs for the prediction model, as well as different configurations of the techniques employed, as training algorithms and new data.

## 7 Conclusions

The floods that occur in the Magdalena River have a great impact on most of the country since they affect not only the economy, but the lives of those who live on its banks that corresponds to 13% of the total population of Colombia. For this reason, it is necessary to find a tool that allows to know the levels of the river in advance and with the least possible error and thus anticipate the natural disaster and develop emergency plans to reduce economic losses and mainly save as many human lives as possible.

In this approach to the problem, it was of great importance to establish the structure of the model considering the different variables that may be involved in the floods of the Magdalena River.

Artificial intelligence has several techniques for the prediction of time series from existing data. In the case of application it is very important to determine the input variables to the model. Between these inputs, the presence or absence of “El Niño” or “La Niña” phenomena, the season of the year in Colombia and the location of the station from which the data comes were important variables in the model.

The performance of the systems with representative samples from various stations were compared in terms of the MSE error. From the results, it is concluded that this Artificial Neural Networks technique is a suitable option to create predictive systems as long as it is not very complex and does not require high processing machine.

As observed in the results, Artificial Neural Networks are the best option in relation to MSE, however, to establish a model based on rules to achieve a better interpretability of the floods, the ANFIS model can be used. In a further work the TSK fuzzy model can be converted to a Mamdani model to get a better description of the relations among inputs and output using fuzzy sets. However, some configurations using ANFIS could not be trained with the available computer system.

The research had time, data, human, financial resources and technological infrastructure limitations. For which only three types of artificial intelligence techniques (ANN, ANFIS, VSM) were analyzed. Nevertheless, it is pertinent to continue with the research including other artificial intelligence prediction techniques such as machine learning, expert systems, Bayesian networks, among others, with the availability of greater resources for the development of the research. For example, a more robust computer system with which a greater number of experimental tests can be done.

## Declarations

### Author contribution statement

Jenny Marcela Moreno, Juan Manuel Sanchez & Helbert Eduardo Espitia: Conceived and designed the experiments; Performed the experiments; Analyzed and interpreted the data; Contributed reagents, materials, analysis tools or data; Wrote the paper.

### Funding statement

This research did not receive any specific grant from funding agencies in the public, commercial, or not-for-profit sectors.

### Competing interest statement

The authors declare no conflict of interest.

### Additional information

No additional information is available for this paper.
